# Diabetic Ketoacidosis at the Onset of Type 1 Diabetes Mellitus Among Children and Adolescents in Jeddah, Saudi Arabia: A Study From the Emergency Department

**DOI:** 10.7759/cureus.24456

**Published:** 2022-04-25

**Authors:** Mawaddah Batwa, Lujain Alharthi, Reem Ghazal, Meaad Alsulami, Rahaf Slaghour, Renad Aljuhani, Abdullah Bakhsh

**Affiliations:** 1 Medicine, Sultan Bin Abdulaziz Humanitarian City, Riyadh, SAU; 2 Medicine, King Abdulaziz University Hospital, Jeddah, SAU; 3 Medicine and Surgery, King Abdulaziz University Faculty of Medicine, Jeddah, SAU; 4 Emergency Medicine, King Abdulaziz University, Jeddah, SAU

**Keywords:** polydipsia, polyuria, cerebral edema, retrospective study, pediatrics emergency, diabetes mellitus type i, diabetic ketoacidosis (dka)

## Abstract

Background: Diabetic ketoacidosis (DKA) is a life-threatening complication of type 1 diabetes mellitus (T1DM) and a leading cause of morbidity and mortality in children. We aim to assess the frequency, clinical characteristics, biochemical findings, and outcomes of DKA at the onset of T1DM in young children and adolescents.

Design and methods: This retrospective cohort study analyzed the medical records of patients ≤ 16 years old seen in the emergency department at King Abdulaziz University Hospital, Jeddah, Saudi Arabia, between April 2015 and June 2019. The severity of DKA was classified according to the International Society for Pediatric and Adolescent Diabetes (ISPAD) criteria.

Results: Out of 207 patients with T1DM, 53 presented with DKA as a new onset. The mean age was 8.51 ± 3.81 years, with the majority being 5-10 years old (52.8%). Polyuria (98.1%), polydipsia (86.8%), weight loss (62.3%), and abdominal pain and vomiting (45.3%) were the most frequent symptoms. Mean random blood glucose was 424.09 ± 108.67 mg/dL and mean venous pH was 7.15 ± 0.36 mmol/L. Of patients, 66% had no associated complications, 24.4% had hypokalemia, 20.8% developed hypoglycemia, and 18.9% developed hyperchloremic metabolic acidosis. One patient had cerebral edema and coma. Based on metabolic acidosis, 24.5% had mild DKA, an equal percentage had severe DKA, and 9.4% had moderate DKA. Of patients, 88.7% were admitted to the pediatric ward and 15.1% to the intensive care unit.

Conclusion: A total of 25% of patients diagnosed with T1DM below the age of 17 years presented with DKA. No permanent disabilities or deaths were reported. Forming a registry dedicated to T1DM is needed to follow up on these patients, especially among school-age children, as well as aid in the development of future research locally.

## Introduction

Diabetes mellitus (DM) is a common chronic disease that affects children and adults worldwide. It is a metabolic disorder characterized by persistent elevation of blood glucose as a result of a defect in insulin secretion, insulin action, or both [1,2]. A study in 2013 concluded that the prevalence of DM in Saudi Arabia (SA) is 20.2% [3]. In 2015, the Ministry of Health in SA reported that 748,605 diabetic patients were admitted to the emergency department [4].

Type 1 diabetes mellitus (T1DM), known as insulin-dependent or childhood-onset diabetes, is the most common chronic disease in children and youths [5]. It is a cell-mediated autoimmune destruction of the insulin-producing beta cells of the pancreas and, therefore, daily administration of exogenous insulin is required [1,5,6]. According to the International Diabetes Federation (IDF), the annual incidence of T1DM is 132,600 cases worldwide, with SA ranking eighth in the number of cases (2,800 cases per year) [5].

A major complication of T1DM is diabetic ketoacidosis (DKA), which is a state of metabolic deterioration as a result of hyperglycemia, acidosis, ketonemia, and ketonuria [7]. It is an acute, life-threatening medical emergency that requires prompt treatment and monitoring for various metabolic abnormalities and observance for complications. About 24% of children with new-onset diabetes presented with DKA [8,9]. The importance of DKA lies in the fact that it is the most common cause of death in diabetic patients, contributing to 83% of diabetes-related deaths as well as inflicting permanent disability in newly diagnosed T1DM in children and adolescents [10-12].

Common symptoms that point to the diagnosis of DKA include polyuria, polydipsia, weight loss and fatigue, rapid shallow breathing, nausea and vomiting, abdominal pain, and altered level of consciousness (LOC). These nonspecific symptoms may be mistaken for other diseases such as pneumonia, asthma, and abdominal conditions [8,13,14].

Complications related to DKA management include cerebral edema, hypokalemia, hypoglycemia, and hyperchloremic acidosis. Cerebral edema is a life-threatening complication of DKA that occurs in about 0.5-1% of all episodes of DKA in children [15,16] and accounts for 60-90% of all DKA deaths [14,17]. Alongside its morbidity and mortality, the economic cost of DKA is high, i.e., an average of $7142 per admission [18,19].

A study in the Al-Baha region in SA estimated that the incidence of T1DM was 355 per 100,000, and 40.8% of the sample presented with DKA at new onset [20]. Another study carried out in Kuwait found that patients who presented with DKA had a longer period of hospitalization [21].

Despite global placement, we lack data that demonstrate the incidence and prevalence of DKA as the first presentation of T1DM in emergency departments. To our knowledge, there are no studies documenting emergency department visits for DKA and their outcomes, thus making it difficult to fully grasp the scope of the problem and reach a better understanding of DKA at the onset of T1DM in SA compared to other countries.

This study aims to assess the frequency, clinical characteristics, biochemical findings, and outcomes of DKA at the onset of T1DM in young children and adolescents at King Abdulaziz University Hospital (KAUH), Jeddah, SA.

## Materials and methods

This is a retrospective cohort study conducted at KAUH, a tertiary center in Jeddah, SA. We reviewed and analyzed medical records and laboratory results of all pediatric patients aged ≤ 16 years, newly diagnosed with T1DM, who presented with DKA as a new onset of the disease to the Department of Emergency Medicine at KAUH. This study was approved by the Institutional Review Board (IRB) of KAUH.

Medical records between April 2015 and June 2019 were reviewed. A total of 207 patients diagnosed with T1DM presented to the emergency department. We included 53 patients who presented with DKA as new onset of the disease in the study.

Patients presenting with hyperglycemia without acidosis, previously diagnosed with T1DM, cystic fibrosis, or hemochromatosis, or who left without receiving treatment were excluded from the study.

We applied the current biochemical criteria for the diagnosis of DKA published by the International Society for Pediatric and Adolescent Diabetes (ISPAD): hyperglycemia (blood glucose > 11 mmol/L; ~200 mg/dL), metabolic acidosis based on severity (mild acidosis: pH < 7.3, bicarbonate < 15 mmol/L; moderate acidosis: pH < 7.2, bicarbonate < 10 mmol/L; and severe acidosis: pH < 7.1, bicarbonate < 5 mmol/L), in addition to ketonemia and ketonuria.

The information extracted included demographic data such as age, gender, and nationality; presenting symptoms; special laboratory findings; and admission and outcome of the patients in terms of developing complications or death.

Data were entered using online Google Forms (Google, Mountain View, CA) and then exported to Microsoft Excel (Microsoft Corporation, Redmond, WA). All statistical analysis was done using IBM SPSS Statistics version 21 (IBM Corp., Armonk, NY). The primary outcome of this study is to determine the frequency of DKA at the onset of T1DM. Our secondary outcomes include the clinical characteristics, biochemical findings, and outcome.

## Results

During the five-year study period, a total of 207 patients aged 16 years old or less were diagnosed with T1DM; 53 (25.6%) of them presented with new-onset DKA. Of patients, 60% were of Saudi nationality.

The mean age was 8.51 ± 3.81 years (9.11 ± 4.05 for boys and 7.84 ± 3.48 for girls), with the majority of the patients being 5-10 years old (52.8%). More than half of the sample (52.8%) were boys, with no significant relation between DKA and either age or gender. The average weight was 31.99 ± 23.02 kg.

Out of all documented symptoms, the most frequent presenting symptoms were polyuria (98.1%), polydipsia (86.8%), weight loss (62.3%), and abdominal pain and vomiting (45.3%), with a mean duration of symptoms of 16.23 ± 17.20 days. Mean random blood glucose and venous pH at diagnosis were 424.09 ± 108.67 mg/dL and 7.15 ± 0.36 mmol/L, respectively. Mean bicarbonate was 12.71 ± 5.96 mmol/L. Other clinical characteristics and laboratory findings are displayed in Table 1.

**Table 1:** Demographics, clinical symptoms, and biochemical findings of DKA DKA, diabetic ketoacidosis; SD, standard deviation; LOC, level of consciousness; RBG, random blood glucose; HbA1C, hemoglobin A1C.

Features	No. (%), mean ± SD
Gender	
Female	25 (47.2)
Male	28 (52.8)
Nationality	
Saudi	32 (60.4)
Non-Saudi	21 (39.6)
Age at presentation (years)	8.51 ± 3.81
Age group	
<5 years	9 (17.0), 2.44 ± 1.24
5-10 years	28 (52.8), 8.11 ± 1.83
>10 years	16 (30.2), 12.63 ± 1.75
Weight in kg	31.99 ± 23.02
Clinical symptoms	
Polyuria	52 (98.1)
Polydipsia	46 (86.8)
Polyphagia	12 (22.6)
Nocturia	18 (34.0)
Weight loss	33 (62.3)
Kussmaul breathing	12 (22.6)
Shortness of breath	17 (32.1)
Abdominal pain	24 (45.3)
Nausea	11 (20.8)
Vomiting	24 (45.3)
Decreased appetite	20 (37.7)
Fatigue	22 (41.5)
Dizziness	18 (34.0)
Altered LOC	7 (13.2)
Duration of symptoms (days)	16.23 ± 17.20
Biochemical findings	
RBG (mg/dL)	424.09 ± 108.67
Venous pH serum (mmol/L)	7.15 ± 0.36
Bicarbonate (mmol/L)	12.71 ± 5.96
Ketonuria	2.64 ± 0.63
Glucose in urine	3.32 ± 0.67
HbA1C	11.94 ± 2.16

Regarding hospital courses, a total of 47 patients were admitted to the pediatric ward (88.7%), while eight patients were admitted to the ICU (15.1%). During the course of management, 66% had no complications; however, hypokalemia, hypoglycemia, and hyperchloremic acidosis occurred in 24.4%, 20.8%, and 18.9% of patients, respectively. Cerebral edema and coma were observed in one patient with severe DKA. The mean duration of hospitalization was 6.40 ± 3.78 days (Table 2).

**Table 2:** Course of hospitalization and treatment-related complications

Outcomes of patients	
Course of hospitalization	
Admitted to the pediatric ward	47 (88.7)
Admitted to the ICU	8 (15.1)
Duration of hospitalization (in days)	6.40 ± 3.78
Treatment-related complications	
Cerebral edema	1 (1.9)
Hypoglycemia	11 (20.8)
Hypokalemia	13 (24.5)
Hyperchloremic acidosis	10 (18.9)
Coma	1 (1.9)
Death	0 (0.0)
No complications	35 (66.0)

Diagnosed children were categorized according to the severity of acidosis. Table 3 shows the comparison between the three groups.

**Table 3:** Comparison between DKA categories DKA, diabetic ketoacidosis; SD, standard deviation; LOC, level of consciousness; RBG, random blood glucose; ICU, intensive care unit.

All, No. (%), mean ± SD	Mild DKA	Moderate DKA	Severe DKA
Frequency	13 (24.5%)	5 (9.4%)	13 (24.5%)
Gender			
Female	8 (61.5)	2 (40.0)	5 (38.5)
Male	5 (38.5)	3 (60.0)	8 (61.5)
Age at presentation (years)	10.00 ± 4.06	8.80 ± 4.44	8.77 ± 3.30
Age groups			
<5 years	1 (7.7)	1 (20.0)	1 (7.7)
5-10 years	7 (53.8)	2 (40.0)	8 (61.5)
>10 years	5 (38.5)	2 (40.0)	4 (30.8)
Clinical symptoms			
Polyuria	13 (100)	5 (100)	13 (100)
Polydipsia	12(92.3)	5 (100)	12 (92.3)
Weight loss	9 (69.2)	3(60.0)	7 (53.8)
Abdominal pain	4 (30.8)	3 (60.0)	9 (69.2)
Vomiting	7 (53.8)	4 (80.0)	8 (61.5)
Altered LOC	2 (15.4)	0 (0.0)	5 (38.5)
Biochemical findings			
RBG (mg/dL)	432.7 ± 153.2	490.6 ± 142.2	448.3 ± 68.0
Venous pH	7.23 ± 0.03	7.15 ± 0.03	6.81 ± 0.55
Serum bicarbonate (mmol/L)	12.2 ± 4.4	12 ± 5.2	8.1 ± 4.8
Ketonuria	2.7 ± 0.7	2.8 ± 0.4	3.1 ± 0.4
Glucose in urine	3.2 ±0.6	3.4 ± 0.5	3.5 ± 0.8
Course of hospitalization			
Admitted to the pediatric ward	12(92.3), p = 0.876	5 (100), p = 0.845	8 (61.5), p = 0.006
Admitted to the ICU	1 (7.7), p = 0.536	0 (0.0), p = 0.659	7 (53.8), p = 0.000
Treatment-related complications			
Cerebral edema	0 (0.0)	0 (0.0)	1 (7.7)
Hypoglycemia	1 (7.7)	0 (0.0)	2 (15.4)
Hypokalemia	5 (38.5)	1 (20)	4 (30.8)
Hyperchloremic acidosis	4 (30.8)	1 (20)	3 (23.1)
Coma	0 (0.0)	0 (0.0)	1 (7.7)
Death/survival	0/13	0/5	0/13

In our study, the majority of the patients presented with mild and severe DKA (24.5% and 24.5%, respectively). Of those presenting with severe DKA, 61.5% were aged 5-10 years old, but no significant difference was found between different age groups (p = ‏0.41). The proportion of girls was higher in mild DKA (61.5%), while in the other categories, the proportion of males was higher, i.e., 60.0% and 61.5% in the moderate and severe DKA, respectively, but no significant relationship between gender and severity was observed.

Polyuria was the main symptom in all patients within the three categories, whereas altered LOC and Kussmaul breathing were significantly higher among patients with severe acidosis (p = 0.019 and p = 0.002, respectively). Mean random blood glucose was higher among patients with moderate DKA, and hypokalemia was more common in mild and severe DKA; however, these associations were not significant.

Regarding admission and hospitalization, there was a significant relationship between severe DKA and admission to ICU and the pediatric ward, and length of hospitalization (p = 0.000, p = 0.006, and p = 0.013, respectively). No deaths were reported among all 53 patients.

## Discussion

The prevalence of DKA at the onset of T1DM varies from 39% to 77% in local regions [20,22,23] of SA compared to the prevalence in our study, which was 25.6%. This lower prevalence may be explained by the availability of many medical facilities and awareness campaigns in our region leading to early detection and diagnosis of T1DM and, thus, a reduction in the incidence of DKA in emergency department visits. Our incidence matched with other developed countries such as the UK (25%), Germany (27%), and the USA (30%) [24].

The mean age at presentation was 8.51 years, with a higher incidence of children aged 5-10 years, accounting for 52.8%, which is consistent with data reported locally and from developing countries (7.3 ± 5.15, 47.9%) [7,9,20,21]. In contrast to a study in Al-Madinah city, where the data were obtained from a maternity and children's hospital, the frequency of children < six years old was higher. This distinction could be related to differences in ethnicity, lifestyles, and socioeconomic status.

The common presenting symptoms of T1DM are polyuria and polydipsia (98.1% and 86.8%, respectively), which is in agreement with other studies [21,25-27]. Abdominal pain and vomiting (45.3%) are more likely to be presented with DKA (34.7% and 47.8%, respectively) [23].

In our study, most children had either mild (24.5%) or severe DKA (24.5%), similar to a study from Kuwait where mild DKA accounted for 42.4%. These results differ from studies in Al-Baha city [20] and Pakistan [7], where moderate cases were higher, 41.3% and 53.1%, respectively, with no association between age and severity of DKA (Table 3).

The percentages of the most common management-related complications according to the ISPAD [14] are hypokalemia (24.5%), hypoglycemia (20.8%), and hyperchloremic acidosis (18.9%). Few studies have illustrated the frequencies of these complications [20,28]. It is essential to point out that these acute complications are frequently seen during management.

A rare complication related to DKA treatment is cerebral edema, which was seen in one patient (1.9%), correlating with other studies, where it ranged from one to three patients [20-22]. All patients who developed complications recovered completely without persistent morbidities. On the other hand, 66% were discharged without complications.

In patients with severe acidosis, we observed a higher incidence rate of admission to the pediatric ward (p = 0.006) and ICU (p = 0.00), with longer durations of hospitalization compared to those in less severe categories (8.62 ± 5.49 vs. 5.53 ± 2.87, p = 0.013). A similar result was observed in Kuwait, where researchers found a negative correlation between pH and length of stay in hospital (p < 0.05) [21].

Despite Kussmaul breathing and altered LOC being less frequent symptoms (22.6% and 13.2%, respectively), a study from Pakistan [6] showed that they are significantly related to severe DKA (p = 0.002, p = 0.019). In terms of the patient outcome, no deaths were reported, similar to results from Al-Baha [20] and Kuwait [29]. In developing and developed countries, the mortality rate ranges are 6-24% [30] and 0.15-0.31% [31].

One of the limitations of the study is that we could not categorize patients whose venous pH results at presentation were not documented. Furthermore, the generalizability of the study is restricted by extracting data from a single center.

We recommend multicenter studies to represent the population accurately and forming a registry dedicated to T1DM patients so that physicians can follow them consistently for glucose in blood and urine, especially among school-age children, as well as to aid in the development of future studies locally.

## Conclusions

The strength of our study lies in the fact that these data were obtained exclusively from emergency visits. We aimed to assess the frequency, clinical and biochemical findings, and outcome of DKA at the onset of T1DM. We found that the prevalence of DKA at the diagnosis of T1DM was 25.6%. Polyuria, polydipsia, weight loss, abdominal pain, and vomiting were common presenting symptoms. The observed mean random blood glucose and venous pH at diagnosis were 424.09 ± 108.67 mg/dL and 7.15 ± 0.36 mmol/L. The higher incidences of DKA were in the mild and severe categories. Although one patient suffered from cerebral edema, no deaths were reported and all patients were discharged without permanent morbidities.
